# Palmitic‐Acid‐Based Hydrophobic Deep Eutectic Solvents for the Extraction of Lower Alcohols from Aqueous Media: Liquid–Liquid Equilibria Measurements, Validation and Process Economics

**DOI:** 10.1002/gch2.201900024

**Published:** 2019-07-26

**Authors:** Rupesh Verma, Tamal Banerjee

**Affiliations:** ^1^ Department of Chemical Engineering Indian Institute of Technology Guwahati Guwahati 781039 Assam India

**Keywords:** ASPEN Plus, COSMO‐SAC, deep eutectic solvents, lower alcohols, NRTL, pseudocomponents, sigma profiles, UNIQUAC

## Abstract

A new, natural, hydrophobic deep eutectic solvent (NADES) based on DL‐menthol and palmitic acid is adopted for the extraction of alcohols from aqueous phase. DL‐menthol is used as a hydrogen bond acceptor and palmitic acid, being a natural organic acid, as a hydrogen bond donor. The synthesis is carried out by the addition of DL‐menthol and palmitic acid in a defined molar ratio. Physical properties of NADES along with water stability are then measured. Liquid–liquid equilibria (LLE) of lower alcohols, namely, DES (1) + lower alcohols (ethanol/1‐propanol/1‐butanol) (2) + water (3) are carried out at *p* = 1 atm and *T* = 298.15 K. LLE results show type‐I phase behavior, where alcohol is preferentially attracted toward DES. The tie lines are then regressed via nonrandom two liquid and universal quasichemical models, which give root mean square deviation (RMSD) in the range of 0.29–0.35% and 0.39–0.75%, respectively. Finally, the quantum‐chemical‐based conductor‐like screening model‐segment activity coefficient is used to predict the tie lines, which gives an RMSD of 2.1–5.2%. A hybrid extractive distillation flowsheet is then used for scale up, process economics, and solvent recovery aspects in ASPEN using DES as a “pseudocomponent.”

## 1 Introduction

Deep eutectic solvents (DES) are new classes of solvent having similar characteristics and physiochemical properties like ionic liquids (ILs). These have thus become a widely used extraction media among researchers and scientists especially due to the higher costs and the complex preparation process associated with ILs. There are certain other factors that set them apart from other solvents such as ease of preparation within the temperature range of 60–80 °C, economical, biodegradable, high boiling point, and low melting point. It also remains in liquid form at room temperature. DESs have low melting point and low lattice energy due to their large, nonsymmetrical ions.^1^, ^2^ Recently, DES is used as azeotrope breakers for lower alcohols and water solution.^3^ Lower alcohols (ethanol, 1‐propanol, and 1‐butanol) are considered as fuel for future generation. Its calorific values are found to be similar to fossil fuel such as gasoline and diesel.^4^, ^5^ Acetone‐butanol‐ethanol (ABE) fermentation is found to be effective for commercial production of bio‐ethanol and bio‐butanol.^6^, ^7^ But alcohols produced by this method are highly aqueous in nature. Separation of lower alcohols is found challenging because of the formation of the azeotrope. Liquid–liquid equilibria (LLE) are found to be suitable for the separation as well as production of lower alcohols up to commercial level.^8^, ^9^, ^10^ In the LLE process, a solvent is used that shows a higher affinity toward alcohols as compared to water.

In available literature, DES being hydrophilic in nature and unstable in water has been reported. However in cases such as alcohol extraction from aqueous solution, hydrophobic solvents are necessary. Hydrophobic DES is known to have favorable solvation properties for both nonpolar and polar compounds.^1^, ^2^, ^11^ It is also found effective for the extraction of organic and inorganic compounds.^12^, ^13^ It is a well‐known fact that chloride‐ion based DESs when heated at high temperatures produce a higher charge density and result in corrosion within the process vessels.^14^ Due to this reason halogen containing DESs are not recommended even if they show high distribution coefficient as well as selectivity.^15^ In such a scenario, a new and cheaper hydrophobic deep eutectic solvent is necessary.

Keeping this in mind the current work reports the synthesis of DES comprising DL‐menthol (hydrogen bond acceptor (HBA)) and palmitic acid (hydrogen bond donor (HBD)) in a 12:1 molar ratio. Further, extraction of ethanol/1‐propanol/1‐butanol (lower alcohols) is proposed via this new DES.^12^, ^15^ In the present study for synthesis of DES, molar ratio of DL‐menthol (HBA) + palmitic acid (HBD) is arrived at 12:1 at *T* = 298.15 K and *p* = 1 atm. This ratio is computed from Conductor‐like Screening Model‐Segment Activity **C**oefficient (COSMO‐SAC) model that involves a solid–liquid equilibrium prediction using the screening charge densities or the COSMO files of HBD, HBA, alcohol, and water. Significant density difference, hydrophobicity, as well affinity toward the alcohols make the current DES effective for breaking the azeotrope between alcohol and water. Formation of H‐bond takes place during synthesis between HBA and HBD. In the later sections, LLE experiments and predictions of tie lines are carried out by nonrandom two liquid (NRTL), universal quasichemical (UNIQUAC), and COSMO‐SAC models . The latter model is based on quantum chemistry calculations. Once the LLE results are available, a scale‐up is required for the separation of lower alcohols from water. A hybrid extraction–distillation process is adopted for such a purpose in order to aid the separation, recovery, and recycle of DES.

## 2 LLE Using DES

In each LLE experiment, a known quantity of water, alcohol, and DES is added in a 30 mL size glass vial. Components are added in such a molar ratio that a heterogeneous region is formed. Parafilm is used as a seal for glass vials to avoid any evaporation loss. The samples are shaken for 12 h, followed by 12 h of settling time so as to achieve equilibrium. Thereafter samples from the extract and raffinate layer are taken via syringes and sent for quantification via ^1^H NMR spectroscopy.

### 2.1 Measurement of LLE

For NMR analysis in the NMR tube (thrift grade, Sigma‐Aldrich), 0.5 mL of NMR solvent is added together with 0.1 mL sample. Parafilm is used to cover up the rubber cap of the NMR tube to avoid any evaporation losses. The NMR tubes are placed in a 600 MHz NMR spectrometer (Make: Bruker) for ^1^H analysis. Reference peak for NMR solvent DMSO‐D6 is recorded at 2.5 ppm. ^1^H NMR is used to calculate the mole fraction of each compound within the extract and raffinate phases. >CH‐ group peak of the DES is found at ≈3.15 ppm in both extract and raffinate phases. Equation (1) is used to calculate the mole fraction of individual component^16^, ^17^ as given below

(1) $$x_{i}=\frac{H_{i}}{\sum_{i=1}^{n}H_{i}}$$

In the above equation, *H_i_* is the peak area due to single H‐atom and *x_i_* is the mole fraction of the component in the mixture.^9^

Figure S5 (Supporting Information) shows the ^1^H NMR spectra and the calculation procedure for the synthetized DES. In NMR spectra, —OH group of DL‐menthol (peak number 15) is 12 times the —OH group of palmitic acid (peak number 14), which implies that the DES exists as a single component or solvent. For water stability analysis, Figures S6 and S7 (Supporting Information) depict the ^1^H NMR of the washed DES (four times to check their hydrophobicity). ^1^H NMR spectra of bottom part (Figure S7, Supporting Information) clearly reflect the absence of DES and confirm the hydrophobic nature of DES. Further Karl Fischer Titrator (Metrohm 787 KF Titrino) is also used for reconfirmation showing an absence of water. The presence of water is observed within the uncertainty range as given by Chapeaux et al.^18^ for ionic liquid mixtures.

## 3 Results and Discussions

### 3.1 Extraction of Lower Alcohols by DL‐Menthol and Palmitic Acid Based Hydrophobic DES

The LLE for the ternary systems namely, DES (1) + lower alcohols (2) + water (3) are measured at *p* = 1 atm and *T* = 298.15 K. **Tables** **1** **–** **3** show the experimental mole fraction of ethanol, 1‐propanol, and 1‐butanol obtained in extract and raffinate phases. Distribution co‐efficient (β) and selectivity (*S*) are used to define the extraction capability of a solvent and are given by Equations (2) and (3) as follows

(2) $$\beta_{\mathrm{alc}}=\frac{x_{\mathrm{alc}}^{\mathrm{DES}}}{x_{\mathrm{alc}}^{\mathrm{Water}}}$$

(3) $$S=\beta_{\mathrm{alc}}/\beta_{\mathrm{water}}$$

**Table 1:** Experimental LLE data for the ternary system, DES (1) + ethanol (2) + water (3) at *T* = 298.15 K and *p* = 1 atm

Extract phase			Raffinate phase			β_ethanol_	Selectivity (*S*)
*x*_DES_ [DL‐menthol+ palmitic acid] (12:1)	*x*_ethanol_	*x*_water_	*x*_DES_	*x*_ethanol_	*x*_water_		
0.652	0.074	0.274	0.007	0.015	0.978	4.933	17.609
0.575	0.135	0.29	0.002	0.043	0.955	3.140	10.339
0.45	0.259	0.291	0.003	0.084	0.913	3.083	9.674
0.359	0.338	0.303	0	0.152	0.848	2.224	6.223
0.305	0.379	0.316	0.003	0.205	0.792	1.849	4.634

Note: Standard uncertainties are *u*(*T*) = 0.01 K, *u*(*x*) = 0.001.

**Table 2:** Experimental LLE data for the ternary system, DES (1) + 1‐propanol (2) + water (3) at *T* = 298.15 K and *p* = 1 atm

Extract phase			Raffinate phase			β_propanol_	Selectivity (*S*)
*x*_DES_ [DL‐menthol+ palmitic acid] (12:1)	*x*_propanol_	*x*_water_	*x*_DES_	*x*_propanol_	*x*_water_		
0.653	0.115	0.232	0.004	0.015	0.981	7.667	32.418
0.576	0.187	0.237	0.001	0.028	0.971	6.679	27.362
0.459	0.316	0.225	0.002	0.043	0.955	7.349	31.192
0.348	0.377	0.275	0.001	0.055	0.944	6.855	23.530
0.243	0.453	0.304	0.001	0.065	0.934	6.969	21.412

Note: Standard uncertainties are *u*(*T*) = 0.01 K, *u*(*x*) = 0.001.

**Table 3:** Experimental LLE data for the ternary system, DES (1) + 1‐butanol (2) + water (3) at *T* = 298.15 K and *p* = 1 atm

Extract phase			Raffinate phase			β_1‐butanol_	Selectivity (*S*)
*x*_DES_ [DL‐menthol+ palmitic acid] (12:1)	*x*_1‐butanol_	*x*_w_	*x*_DES_	*x*_1‐butanol_	*x*_w_		
0.61	0.115	0.275	0.002	0.006	0.992	19.167	69.139
0.558	0.179	0.263	0.0024	0.0106	0.987	16.887	63.374
0.457	0.291	0.252	0.001	0.014	0.985	20.786	81.246
0.329	0.394	0.277	0.0015	0.021	0.9775	18.762	66.209
0.261	0.453	0.286	0.001	0.026	0.973	17.423	59.275
0.203	0.494	0.303	0.001	0.034	0.965	14.529	46.274

Note: Standard uncertainties are *u*(*T*) = 0.01 K, *u*(*x*) = 0.001.

Here, mole fraction of alcohols in extract and raffinate phases is given by $x_{\mathrm{alc}}^{\mathrm{DES}}$ and $x_{\mathrm{alc}}^{\mathrm{Water}}$, respectively. The distribution coefficient is related with amount of solvent required for the effective separation. On the other hand selectivity refers how much the solvent (DES) is selective enough for the extraction of alcohol from its aqueous solution. Tables 1, 2, 3 report the selectivity (*S*) and distribution coefficient (β) for ethanol, 1‐propanol, and 1‐butanol. For 1‐butanol, distribution co‐efficient is found to be between 14.5 and 19.2, while for 1‐propanol and ethanol it is in the range of 6.7–7.7 and 1.8–4.9, respectively. Overall the selectivity value must be greater than unity for the liquid–liquid extraction process. The selectivity of 1‐butanol is obtained in the range of 46.2–81.2, while for 1‐propanol and ethanol it varies from 21.4–32.4 and 4.6–17.6, respectively (Tables 1, 2, 3). Table 3 shows a higher value of selectivity for 1‐butanol as compared to ethanol and 1‐propanol. This makes DES to be a preferred solvent to extract 1‐butanol.

**Figure 1:**
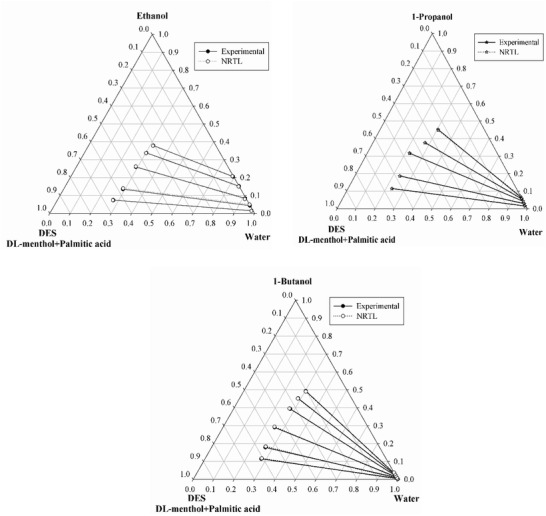
Experimental and NRTL predicted tie lines for the ternary system DES (1) + ethanol/1‐propanol/1‐butanol (2) + water (3) system at *T* = 298.15 K and *p* = 1 atm (DL‐menthol:palmitic acid = 12:1).

LLE data are needed to predict the separation process; hence, activity coefficient based on excess Gibb's free energy models, NRTL, and UNIQUAC are used to regress the experimental data by predicting the binary interaction parameters.^19^, ^20^ **Figure** **1** shows the ternary plots, in which experimental data are correlated with the NRTL model. In the ternary diagram (Figure 1) slope of the 1‐butanol is observed to be higher in comparison to ethanol and 1‐propanol. This shows that the new hydrophobic DES has potential to extract lower alcohols from their aqueous solution. For all the systems the binary interaction parameters, objective function, and root mean square deviation (RMSD) values are given in **Table** **4**. Using NRTL model, RMSD values obtained are 0.29%, 0.30%, and 0.35% for ethanol, 1‐propanol, and 1‐butanol systems, respectively (Table 4). Similarly, RMSD values obtained via the UNIQUAC model are 0.75%, 0.57%, and 0.39% for systems involving ethanol, 1‐propanol, and 1‐butanol, respectively (Table 4). **Tables** **5** **–** **7** show the literature data for a comparison of distribution coefficients and selectivities that are used for the separation of lower alcohols using various DES and ILs. It is clear that the selectivities for choline chloride and tetramethylammonium chloride as hydrogen bond acceptor^12^ are comparable to our DES, which is based on DL‐menthol. The obtained results also show that new DES is found effective as compared to the ILs for the extraction of ethanol, 1‐propanol, and 1‐butanol (Tables 5, 6, 7).^21^, ^22^, ^23^

**Table 4:** NRTL and UNIQUAC interaction parameters for ternary systems at *T* = 298.15 K and *p* = 1 atm

*i*–*j*	NRTL model parameters				UNIQUAC model parameters			
	τ_ij_	τ_ji_	*F* ^a)^	%RMSD^a)^	*A*_ij_/*K*	*A*_ji_/*K*	*F* ^a)^	%RMSD^a)^
DES (1) + ethanol (2) + water (3)								
1–2	2.26	11.06	−1.71 × 10^−4^	0.29	326.25	433.69	−3.2 × 10^−4^	0.75
1–3	20.00	2.18			505.94	998.96		
2–3	17.18	−0.27			399.3	−86.571		
DES (1) + propanol (2) + water (3)								
1–2	19.97	20.00	−2.7 × 10^−4^	0.30	232.48	317	−9.87 × 10^−4^	0.57
1–3	3.02	1.04			463.98	169.59		
2–3	2.48	6.50			517.71	13.666		
DES (1) + 1‐butanol (2) + water (3)								
1–2	2.93	8.17	−4.51 × 10^−4^	0.35	152.85	395.49	−4.7 × 10^−4^	0.39
1–3	−0.13	−2.69			377.82	58.647		
2–3	19.01	14.02			394.97	76.301		

^a)^ Procedure as explained by Verma et al.^9^, ^10^ where $RMSD={\left[\sum_{k=1}^{m}\;\sum_{i=1}^{c}\;\sum_{I=l}^{II}\frac{{\left(x_{ik}^{l}-{\hat{x}}_{ik}^{l}\right)}^{2}}{2mc}\right]}^{1/2}$, where *m* and *c* refer the number of tie lines and the number of components, respectively. Experimental and predicted mole fraction is referred as $x_{ik}^{l}$and ${\hat{x}}_{ik}^{I}$ for component *i* in the *k*th tie line for phase *l*, respectively.

**Table 5:** Comparison of distribution coefficients and selectivities for ethanol extraction in aqueous media using conventional solvents, ionic liquids, and DESs

System	Distribution coefficient	Selectivity	References
DES (DL‐menthol/palmitic acid)	0.52	9.6	This work
DES [glycerol/choline chloride with molar ratios (4:1)]	0.811	21.9	Rodriguez et al.^12^
DES [glycerol/choline chloride with molar ratios (2:1)]	0.618	15.2	Rodriguez et al.^12^
DES [glycerol/tetramethylammonium chloride with molar ratios (4:1)]	0.643	13.3	Rodriguez et al.^12^
DES [glycerol/tetramethylammonium chloride with molar ratios (2:1)]	0.725	14.7	Rodriguez et al.^12^
[TDTHP][Phosph]*	0.83	5.1	Neves et al.^21^
[TDTHP][Deca]*	0.82	4.9	Neves et al.^21^
[TDTHP]Cl*	0.88	6.6	Neves et al.^21^
[TDTHP][CH_3_SO_3_]*	0.82	4.6	Neves et al.^21^
[TDTHP]Br*	0.70	8.4	Neves et al.^21^
[TDTHP][N(CN)_2_]*	0.51	6.8	Neves et al.^21^
[TDTHP][Tf_2_N]*	0.31	2.0	Neves et al.^21^
[BMIM][Tf_2_N]*	0.15	7.5	Cháfer et al.^50^

* Abbreviations: [TDTHP][Phosph]: Tetradecyltrihexylphosphonium bis(2,4,4‐trimethylpentyl) phosphinate[TDTHP][Deca]: Tetradecyltrihexylphosphonium decanoate[TDTHP]Cl: Tetradecyltrihexylphosphonium chloride[TDTHP][CH_3_SO_3_]: Tetradecyltrihexylphosphonium methane sulfonate[TDTHP]Br: Tetradecyltrihexylphosphonium bromide[TDTHP][N(CN)_2_]: Tetradecyltrihexylphosphonium dicyanimide[BMIM][Tf_2_N]: 1‐butyl‐1‐methylpyrrolidinium bis(trifluoromethyl sulfonyl) imide[TDTHP][Tf_2_N]: Tetradecyltrihexylphosphonium bis(trifluoromethyl sulfonyl) imide[Bmim][Tf_2_N]: 1‐butyl‐1‐methylpyrrolidinium bis(trifluoromethylsulfonyl)imide.

**Table 6:** Comparison of distribution coefficients and selectivities for propanol extraction in aqueous media using conventional solvents, ionic liquids, and DESs

System	Distribution coefficient	Selectivity	References
DES	2.2	31.19	This work
[BMP][Tf_2_N]*	0.37	19.8	Cháfer et al.^51^
[TDTHP][Phosph]*	1.37	88.5	Bharti et al.^5^

* Where abbreviations: [BMP][Tf_2_N]: 1‐butyl‐1‐methyl‐pyrrolidinium bis(trifluoromethylsulfonyl)imide;[TDTHP][Phosph]:Tetradecyltrihexylphosphonium bis(2,4,4‐trimethylpentyl)phosphinate.

**Table 7:** Comparison of distribution coefficients and selectivities for 1‐butanol extraction in aqueous media using conventional solvents, ionic liquids and DESs

System	Distribution coefficient	Selectivity	References
DES	3.41	81.25	This work
[Im_10,1_][TCB]*	3.2	100	Heitmann et al.^22^
[P_6,6,6,14_][TCB]*	2.0	500	Heitmann et al.^22^
[Im_8,1_][FAP]*	0.8	420	Heitmann et al.^22^
[Im10,1][Tf_2_N]*	5.7	90	Nann et al.^23^
[Mo10,1][TCB]*	4.8	70	Nann et al.^23^
[Mo10,1][Tf_2_N]*	2.1	99.7	Nann et al.^23^
[Bmim][Pf_6_]*	0.74	21.0	Ha et al.^52^
[Hmim][Pf_6_]*	0.97	37.5	Ha et al.^52^
[Omim][Pf_6_]*	1.11	49.2	Ha et al.^52^
[Bmim][Tf_2_N]*	1.03	39.1	Ha et al.^52^
[Hmim][Tf_2_N]*	1.25	66.1	Ha et al.^52^
[Omim][Tf_2_N]*	1.37	78.9	Ha et al.^52^
[Hmim][TfO]*	0.90	2.6	Ha et al.^52^
[Omim][TfO]*	1.03	3.5	Ha et al.^52^
[Pmim][TfO]*	1.05	4.9	Ha et al.^52^
[HMIM]BF_4_]*	0.90	3.9	Ha et al.^52^
[OMIM][BF_4_]*	2.18	12.2	Ha et al.^52^
[Hmim][Tf_2_N]*	1.11	120.0	Garcia et al.^53^
Cyphos 104*	9.21	55.0	Garcia et al.^53^
[MTOAOct]*	11.29	49.0	Garcia et al.^53^
[TDAMCH]*	8.49	130.0	Garcia et al.^53^
[TOAMNaph]*	21.00	274.0	Garcia et al.^53^

* Where abbreviations: [Im_10,1_][TCB]: (1‐decyl‐3‐methyl‐imidazolium tetracyanoborate); [P_6,6,6,14_][TCB]: (Trihexyltetradecylphosphonium tetracyanoborate); [Im_8,1_][FAP]: (1‐decyl 3‐methylimidazolium tris(pentafluoroethyl)trifluorophosphate); [Im_10,1_][Tf_2_N]: (1‐decyl‐3‐methyl‐imidazolium bis(trifluoromethylsulfonyl)imide); [Mo_10,1_][TCB]: (4‐decyl‐4‐methyl‐morpholinium tetracyanoborate); [Mo_10,1_][Tf_2_N]: (4‐decyl‐4‐methyl‐morpholinium bis(trifluoromethylsulfonyl)imide); [Bmim][Tf_2_N]: (1‐butyl‐1‐methylpyrrolidinium bis(trifluoromethylsulfonyl)imide; [Hmim][Tf_2_N]: Tetradecyltrihexylphosphonium bis(trifluoromethylsulfonyl)imide; [Omim][Tf_2_N]: (1‐butyl‐1‐methylpyrrolidinium bis(trifluoromethylsulfonyl)imide); [Hmim][TfO]: 1‐hexyl‐3‐ methylimidazoliu trifluoromethanesulfonate; [Omim][TfO]: 1‐methyl‐3‐octylimidazolium trifluoromethanesulfonate; [Pmim][TfO]: 1‐ phenylpropyl‐3‐methylimidazolium trifluoromethanesulfonate; [HMIM]BF_4_]: 1‐hexyl‐3‐ methylimidazoliu tetrafluoroborate; [OMIM][BF_4_]: 1‐methyl‐3‐octylimidazolium tetrafluoroborate; [Hmim][Tf_2_N]: 1‐hexyl‐3‐methylimidazolium bis(trifluoromethylsulfonyl)imide; Cyphos 104: Tetradecyl(trihexyl) phosphonium bis‐2,4,4‐trimethylpentyl‐phosphinate; [MTOAOct]: Methyltrioctylammonium octanoate; [TDAMCH]: Ttetrakis(decyl) ammonium 1‐methyl‐1‐cyclohexanoate; [TOAMNaph]: Tetraoctylammonium 2‐methyl‐1‐naphthoate.

### 3.2 DES Definition in ASPEN

Once the LLE results are available a need is felt to use these solvents in a hybrid extraction and distillation flowsheet. However as DESs are not available in ASPEN database, we require to insert their properties via “pseudocomponent” routine in ASPEN. For this to happen it requires the sigma profile of HBA, HBD, water, and alcohol. The next few paragraphs discuss the sigma profile of these components so that the DES can be used as an individual entity in ASPEN. Initially the molecular geometries were fully optimized by using “Gaussian09” software at B3LYP/6‐31G* level of theory. After the geometry optimization, the COSMO file was generated using the final optimized structure using the keyword “SCRF = COSMORS.” The first step of COSMO‐SAC calculation is to estimate the sigma profiles (i.e., the screening charge densities) of each species. The detailed description of COSMO‐SAC theory is given in our previous work^16^, ^24^, ^25^ and hence is not discussed here.

**Figure 2:**
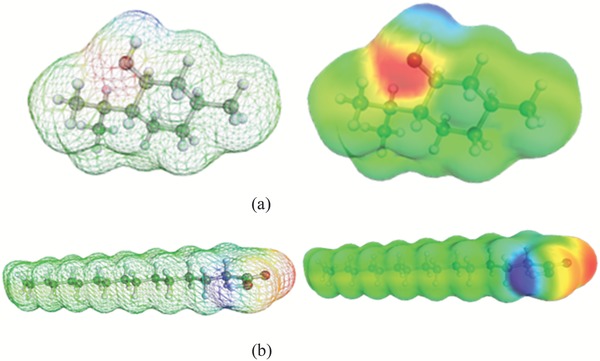
COSMO surfaces of a) DL‐menthol (HBA) and b) palmitic acid (HBD) used for DES syntheses.

**Figure** **2** shows the COSMO surfaces of DL‐menthol (HBA) and palmitic acid (HBD) molecules. Surface color indicates screening charge density with its respective magnitude.The middle column of Figure 2 also shows the segmented regions. While the blue regions have negative screening charge values, green regions indicate neutral and red region positive values. This is true as the COSMO induced screening charges are opposite in nature due to their inherent charge. The red color here represents the values of screening charge densities that are positive in nature while the blue surface represents negative values. This is a basic theory on which the COSMO model depends and applies to all compounds or groups. For DL‐menthol, the red surfaces denote the oxygen atom of the —OH radical while atom indicated by blue color represents the hydrogen atom of the —OH functionality. In a similar manner, within palmitic acid, the terminal oxygen and hydrogen atoms, i.e., within the —COOH group represent the red and the blue surfaces, respectively. The remaining hydrogens or the backbone of the acid are green indicating the nonpolar regions of the acid.

**Figure 3:**
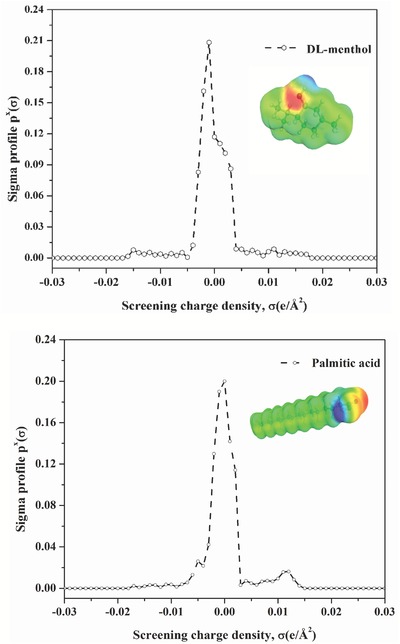
Sigma profile and COSMO segmented surface of HBA and HBD molecules.

**Figure 4:**
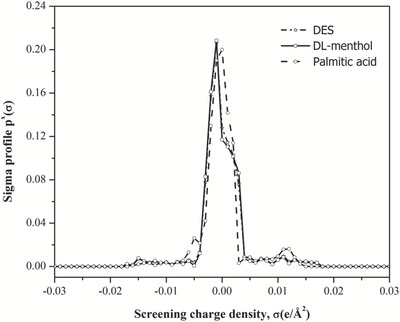
COSMO segmented surface for the menthol‐based DES.

Sigma profiles for the HBD, HBA, as well as DES are shown in **Figures** **3** and **4**. Equation (4) is used to calculate the sigma profile of the DES using a combination of sigma profile of HBA (DL‐menthol) and HBD (palmitic acid) given as below

(4) $$p_{\mathrm{DES}}\left(\sigma \right)=p_{\mathrm{HBA}}\left(\sigma \right)+p_{\mathrm{HBD}}\left(\sigma \right)=f_{\mathrm{HBA}}p_{\mathrm{HBA}}\left(\sigma \right)+f_{\mathrm{HBD}}p_{\mathrm{HBD}}\left(\sigma \right)$$

Here *f*_HBA_ and *f*_HBD_ are the respective molar ratio (i.e., 12:1) of HBA and HBD used for synthesizing the DES. We are now in a position to define the DES in ASPEN. For this we require the molecular weight, density, boiling point, viscosity, COSMO cavity volume term, and sigma profiles. For boiling point, the Lydersen−Joback−Reid method is used to calculate the normal boiling point of DES^26^, ^27^ as given below

(5) $$T_{b}=198.2+\sum n_{i}\Delta T_{bMi}$$

Here, normal boiling temperature of DES is *T*_b_ (K), *n_i_* represents the frequency of molecular group and Δ*T*_b_ *_Mi_* is the boiling point of corresponding group. The normal boiling point of the new DES is given in **Table** **8** and is found to be 545.80 K as calculated via Equation (5).

**Table 8:** Compound name, solubility, boiling point (B.P.), purities, and source of the chemicals used in this work (standard uncertainties *u* are *u*(*T*) = 0.01 K, *u*(ρ) = 0.001 kg m^−3^, and *u*(MP) = ± 1 K)

Compound name	Solubility in water at *T* = 293.15 K	MP [K]	BP [K]	Pure component density [g cm^−3^] at *T* = 293.15 K	Purity (mass fraction)	Manufacturer
DL‐menthol	0.420 gm L^−1^	307.15	487.75	0.890	≥95%	Sigma‐Aldrich, Germany
Palmitic acid	Insoluble	335.15	352.0	0.853	≥97%	Tokyo Chemical Industry, Japan
Ethanol	Infinite	–	351.39	0.789	≥99%	Merck, India
1‐Propanol	Infinite	–	371.15	0.803	≥99%	Merck, India
1‐Butanol	75 gm L^−1^	–	390.85	0.810	≥99%	Merck, India
DES (DL‐menthol:palmitic acid: 12:1)	NM^a)^	296.49^b)^	545.80^c)^	0.890^d)^	≥99%^e)^	This work

Note: All the properties are as per manufacturer specification except for DES.

^a)^ Not measured

^b)^ DSC

^c)^ Joback method^26^

^d)^ Densitometer (DMA 4500, Anton Paar)

^e)^ ^1^H NMR.

### 3.3 Hybrid Extraction Distillation

**Figure 5:**
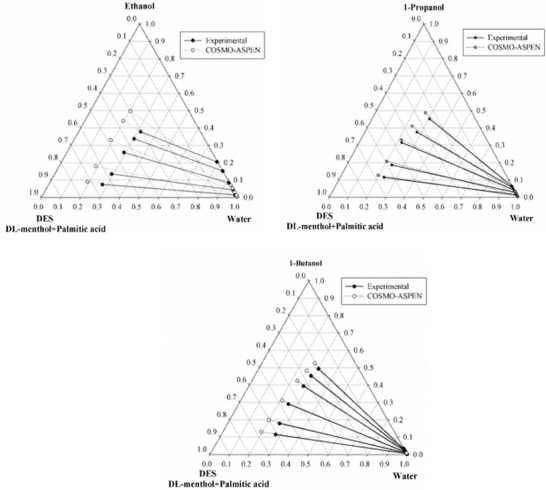
Experimental and COSMO‐SAC predicted tie lines for the ternary system: DES (1)‐alcohol (2)‐water (3) at 298.15 K and 1 atm.

Once the liquid−liquid equilibria and the DES are included within the ASPEN database a scale‐up for the separation of lower alcohols needs to be performed. Commercial software such as ASPEN Plus V8.8 is conventionally used for understanding the process economics of any separation process. The separation of lower alcohols through a hybrid extraction system is already well known.^28^, ^29^ This is also in line with an earlier work^30^ where the hybrid separation processes are reported to be efficient for reducing the energy intensive step of distillation. In the hybrid extraction–distillation process, extractor is operated at ambient temperature and atmospheric pressure. It implies, within the extractor column there is no requirement of additional energy. It shows significant savings in the total annual cost (TAC) as compared to explicit extractive distillation.^49^, ^50^ Initially the tie lines have been predicted using the COSMO based model in ASPEN so as to benchmark the experiments. **Figure** **5** shows the experimental and COSMO‐SAC predicted tie lines for all the systems. The ternary tie lines for DES are depicted for both COSMO‐ASPEN and our in‐house COSMO‐SAC code.^8^ Tie lines that are generated by the COSMO‐SAC model approximately match with the experimental tie lines. RMSD values obtained via the COSMO‐SAC model are 5.2%, 1.6%, and 2.1% for systems containing ethanol, 1‐propanol, and 1‐butanol, respectively. As observed the average RMSD is found to be lesser for the NRTL model. This is expected as COSMO‐SAC^20^, ^31^ is predicted using a statistical mechanical framework while in NRTL,^31^ binary interaction parameters are regressed using the experimental data.

**Figure 6:**
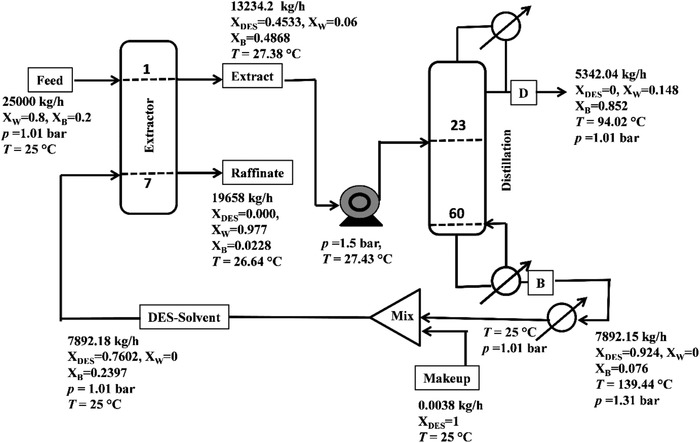
Hybrid extraction–distillation process flow sheet for the separation of 1‐butanol using DES as a solvent.

Extraction of 1‐butanol using DES as a solvent is carried out at *p* = 1 atm and *T* = 25 °C. Feed for simulation is taken 1‐butanol 0.2 w/w and water 0.8 w/w, which is similar as per the reported LLE experiments. The 1‐butanol feed rate is taken as of 5000 kg h^−1^. The entire flowsheet (**Figure** **6**) is optimized as per our previous work.^10^ This includes minimizing the extractor cost using an optimized solvent flow rate so as to further reuse and recycle the solvent.^29^, ^32^, ^33^ For optimizing the extractor, both “Sensitivity Analysis” and “Design Spec” are used for the recovery of 99.99% 1‐butanol from the extract stream by keeping DES (solvent) flow rate as the manipulated variable for a particular number of stages. Extract stream of the extractor is now given as a feed in the distillation column by keeping distillate rate and reflux ratio as the manipulated variable. A feed pump is used to increase the pressure of distillation column feed stream. “Design Spec” is then used in the distillation column for fixing the desired purity of 1‐butanol in the distillate stream. While applying the “Design Spec” in the distillation column, reflux ratio is varied from 0.01 to 100, while the flow rate of distillate varies from 1000 to 6000 kg h^−1^. Feed stage, total number of stage, or pump pressure is varied to make the simulation converge. TAC of the distillation column that contains the capital cost as well as energy cost is calculated while varying the number of stages.^28^, ^34^, ^35^ Optimum TAC is found where the combined TAC of extractor and distillation column is minimum.^29^

**Figure 7:**
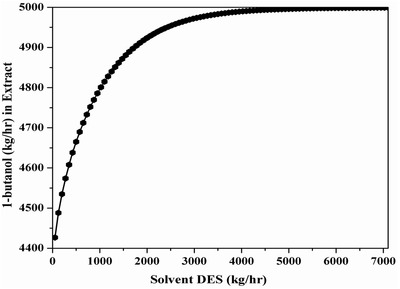
Optimal DES solvent flow rate with 1‐butanol via “sensitivity analysis.”

The optimal results show seven equilibrium stages in the extractor and 60 stages for distillation column. **Figure** **7** shows the sensitivity results for a 100% recovery of 1‐butanol with varying solvent flow in the extract stream. As observed, required flow rate of the solvent is 6000 kg h^−1^ for 0.999 w/w recovery of 1‐butanol in the extractor (Figure 7). In this case also, “Design Spec” is used by keeping the mass fraction of 1‐butanol in the distillate as 0.852. This is due to the strict convergence criteria adopted in the extractor design. Overall a 4.84 reflux ratio along with a 2.93 m column diameter is achieved. **Table** **9** shows 1‐butanol recovery for different streams. 1‐butanol 99.99% (by weight) as extracted with a weight fraction of ≈0.852 w/w. The raffinate stream is dominated by water in the extractor column and this is similar to our experimental results (as shown in Table 3). **Table** **10** shows the TAC of hybrid extractive distillation for the extraction of 1‐butanol in which DES is used as a solvent for a feed of 25 000 kg h^−1^ [water = 0.8, BtOH = 0.2 w/w]. It is observed that DES is the most effective in terms of solvent requirement. From optimization the required solvent flow rate is 6000 kg h^−1^ with a reflux ratio of 4.84 and a reboiler duty of 8.44 × 10^6^ W. This is almost five times less as compared to the conventional solvent mesitylene.^10^

**Table 9:** Stream results for 1‐butanol recovery using DES as a solvent

Stream name*	Feed	DES‐solvent	Extract	Raffinate	*D*	*B*	Make‐up
Component mass flow [Kg h^−1^]							
DES	0.000	5999.95	5999.95	0.0038	0.000	5999.95	0.0038
1‐Butanol	5000.00	1892.23	6443.63	448.599	4551.42	1892.21	0.00
Water	20 000.00	0.00	790.615	19 209.4	790.615	0.00	0.00
Component mass fraction							
DES	0.000	0.7602	0.4533	0.000	0.000	0.7602	1.000
1‐Butanol	0.200	0.2397	0.4868	0.0228	0.852	0.2397	0.000
Water	0.800	0.000	0.060	0.977	0.148	0.000	0.000
Mass flow (kg/h)	25 000.00	7892.18	13 234.2	19 658	5342.04	7892.15	0.0038
Volume flow [lpm]	435.242	161.845	276.622	331.619	115.093	181.195	0.00
*T* [°C]	25.00	25.00	27.38	26.64	94.02	139.44	25.00
P [bar]	1.01	1.01	1.01	1.01	1.01	1.31	1.01
Molar enthalpy [cal mol^−1^]	−68 782.21	−58 136.69	−68 037.9	−68 281.74	−71 310.07	−50 167.3	−43 900.69
Molar entropy [cal mol^−1^ K^−1^]	−44.12767	−180.0307	−124.5664	−39.35165	−84.66176	−158.0951	−214.1626

Note: Optimal results: Extractor column: *P* = 1 atm, *T* = 25 °C, *N*_Extractor_ = 7

Distillation column: *N*_Distillation_ = 60, *N*_feed_ = 23; distillate rate: 5342.04 kg h^−1^; reflux ratio: 4.84; *D*_Distillation_: 2.38 m; reboiler heat duty = 8444.872 KW.

**Table 10:** Overall comparison of DESs as well as mesitylene for the extraction of 1‐butanol

Solvent name	DES	Solvent_Dist.Col._ [kg h^−1^]	0
Feed flow [kg h^−1^] [W = 0.8, Bt = 0.2 w/w]	25 000	Reboiler duty [kw]	8444.87
Solvent required [kg h^−1^]	5999.95	Energy (10^3^ $ year^−1^)	1131.67
RR	4.84	Capital (10^3^ $ year^−1^)	2123.62
*N_T_* extractor	7	TAC_Dist‐Col_* (10^6^ $ year^−1^)	1839.54
*N_T_* Dist. Col.	60	TAC_Ext‐Col_** (10^3^ $ year^−1^)	9.925
*N_F_* Dist. Col.	23	Pump capital cost*** (10^3^ $ year^−1^)	9.473
*D*(m) Dist. Col.	2.38	Pump energy cost*** (10^3^ $ year^−1^)	0.678
Recovered BuOH _Dist Col._			
[kg h^−1^]	4551.42	Cooling water cost*** (10^3^ $ year^−1^)	2.907
TAC overall****(10^6^ $ year^−1^)	1.862		

Note: Based on the methodology given by *Luyben^28^; **Seider et al.^32^; ***Pathak et al.^54^; ****Chen et al.^29^

## 4 Conclusions

In this study, a new natural hydrophobic deep eutectic solvent (NADES) with the combination of DL‐menthol (HBA) and palmitic acid (HBD) is reported. Physicochemical and thermal stability of synthesized DES that includes density, viscosity, melting point, and thermal degradation temperature are reported in the temperature range from 293.15 to 353.15 K. Thereafter extraction of lower alcohols (ethanol/propanol/butanol) is carried out using the DES from aqueous phase. The obtained results are measured and reported by ternary tie line plots through the NRTL, UNIQUAC, and COSMO‐SAC models. Finally a hybrid extraction–distillation is proposed for the scale‐up of the process. It consists of an extractor column and a distillation column for the solvent recovery analysis. An economic consideration with respect to TAC is also attempted. The recovery of 1‐butanol is obtained as ≈91% w/w with a solvent/feed ratio of 0.22 using DES as a solvent. It required a solvent flow rate of ≈6000 kg h^−1^, which was found to be lower than the conventional solvent, i.e., mesitylene. The current study shall integrate the experimental data in laboratory scale with the scale‐up using the TAC as the deciding factor.

## 5 Experimental Section

*Chemicals and Materials*: Table 8 shows the chemicals, purity, solubility, boiling point, melting point, and source. Analytical grade chemical was used without purification. ^1^H NMR spectroscopy was used for the conformation of purities of DL‐menthol, palmitic acid, ethanol, 1‐propanol, and 1‐butanol. The analysis indicated negligible impurities. DMSO‐d6 ≥ 99.8% (dimethyl sulfoxide‐d6) was used as an NMR solvent as supplied by Merck, Germany and was used as received.

*Synthesis of Hydrophobic DES*: In this study, mixture of DL‐menthol and palmitic acid was prepared in such a manner that it remained as a stable liquid at room temperature.^9^, ^36^ Before its preparation, quantum chemical based COSMO‐SAC model was used to predict the molar ratio of HBD and HBA.^37^, ^38^ The application of COSMO‐SAC in areas like distillation, extraction, and absorption is well known.^37^, ^38^, ^39^, ^40^, ^41^, ^42^, ^43^, ^44^ The preparation can start after obtaining the optimum ratio of DL‐menthol and palmitic acid. The simplified form of solid–liquid equilibrium problem as predicted and initiated by the COSMO‐SAC model is given below^16^, ^45^, ^46^

(6) $$\ln\left(x_{s}^{L}\gamma_{s}^{L}\right)=-\frac{\Delta_{\mathrm{fus}}H_{s}}{RT_{s,\mathrm{fus}}}\left(1-\frac{T_{s,\mathrm{fus}}}{T}\right)$$

**Figure 8:**
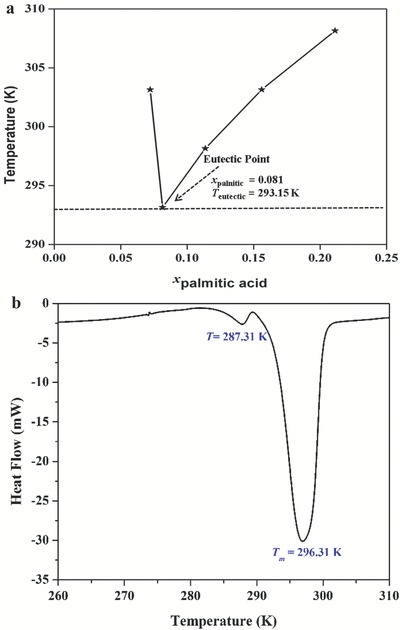
a) COSMO‐SAC prediction of eutectic point and temperature for DES based on DL‐menthol and palmitic acid (12:1). b) Differential scanning calorimetry (DSC) of DL‐menthol and palmitic acid (12:1) based DES.

Here $x_{s}^{L}$ is mole fraction of palmitic acid in DL‐menthol solution. Δ_fus_ *H*_s_ and *T*_s,fus_ are the heat of fusion and melting temperature of the palmitic acid, respectively. *T* (K) is the equilibrium temperature and *R* is the ideal gas constant. Here DL‐menthol is considered as the liquid phase as it has a lower melting point. $\gamma_{s}^{L}$ is the activity coefficient of palmitic acid in DL‐menthol. In the right part of Equation (6), the pure component parameters of the solid solute (palmitic acid) are required. The properties such as heat of fusion and melting point of pure components are shown in **Table** **11**. **Table** **12** shows the COSMO‐SAC prediction and experimental validation for the eutectic compositions and corresponding temperatures for the DL‐menthol and palmitic acid based DES. A direct correspondence with respect to the melting point, namely, 293.15 and 296.31 K was compared and observed between COSMO‐SAC prediction (**Figure** **8**a) and differential scanning calorimetry (DSC) measurements (Figure 8b), respectively. From the prediction, the eutectic point (Figure 8a and Table 12) corresponded to a mole ratio of 12:1 for DL‐menthol + palmitic acid (0.919/0.081). A higher ratio might be primarily due to the enhanced and longer chain of the organic acid leading to higher steric hindrance. Based on the COSMO‐SAC predictions mole ratio of HBA (DL‐menthol):HBD (palmitic acid) was chosen 12:1 for the synthesis of DES.

**Table 11:** Phase Transition Properties for hydrogen bond donor and acceptor

Name of the comp.	*T*_m_ [K]	Δ*H*_f_ [kJ mole^−1^]	Reference
Menthol	308.8	11 000	Corvis et al.^48^
Palmitic acid	336.84	51 020	Pontes et al.^49^

**Table 12:** Coordinates of the eutectic points as predicted with the COSMO‐SAC model

System	Experimental		COSMO‐SAC	
	*x*_DL‐menthol_	*T*/K	*x*_DL‐menthol_	*T*/K
DL‐menthol + palmitic acid (12:1)	0.923	296.31	0.919	293.15

**Figure 9:**
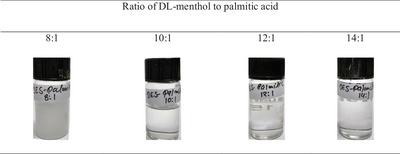
Formation of DES with different molar ratio of DL‐menthol to palmitic acid.

The preparation procedure was carried out in the same manner as our earlier work,^9^ hence is not discussed here. The temperature was maintained 50 °C for first 15 min and further 80 °C for next 1 h. The temperature was maintained at 80 °C because the melting point of palmitic acid was around 62 °C (Table 8). **Figure** **9** shows the visual observation of the synthesized sample at molar ratios of 8:1, 10:1, 12:1, and 14:1 using DL‐menthol + palmitic acid. Crystals were observed below the molar ratio 8:1, while 10:1 was not found to be stable for a long period of time. However ratios greater than 10:1 increased this stability and gave a clear solution. For economical reason, ratios higher than 12:1 were avoided.

*Physicochemical and Thermal Properties of DES*: The next step related to the measurement of the physicochemical properties of DESs. Physicochemical properties play a very important role in designing the process equipment, piping, and pumping units. The density and viscosity were measured in the temperature range 293.15–353.15 K using a densitometer (Anton Paar, DMA4500) and a rheometer (make: Anton Paar, model: Physica MCR 301), respectively. It was seen that the density of the new DES was less than unity (Figure S1, Supporting Information), while the viscosity reached a value of 0.01 Pa s at high temperatures (Figure S2, Supporting Information). DSC temperature calibration was done by indium standard material. The heat flow was calibrated by the melting and heat of fusion of the indium standard where the values of melting point and heat of fusion were 156.34 °C and 28.42 J g^−1^, respectively. The calibration was done at a heating rate of 1 °C min^−1^. The DSC measurements (Figure S3, Supporting Information) were carried out on a DSC STAR^e^ System Mettler Toledo Instrument using 40 µL aluminum crucibles with capillary hole under inert gas (N_2_). This helped to prevent the accumulation of gases, avoid explosions, and remove any volatile chemicals that may be present at atmospheric condition. The melting point of the DES was found to be 296.31 K, which was lower than the melting point of either HBA or HBD. The thermal degradation temperature was performed by a TGA (TG209 F1, Libra, NETZSCH, Germany) under nitrogen atmosphere at a heating rate of 10 °C per minute (Figure S4, Supporting Information).^9^, ^47^ The DES was found to be thermally stable till temperatures as high as ≈400 K, which implied that it could be used an excellent extraction media.

## Conflict of Interest

The authors declare no conflict of interest.

## Supporting information

Supplementary
