# Supplementary material for: Palmitic‐Acid‐Based Hydrophobic Deep Eutectic Solvents for the Extraction of Lower Alcohols from Aqueous Media: Liquid–Liquid Equilibria Measurements, Validation and Process Economics
Source: Glob Chall. 2019 Jul 26;3(11):1900024. doi: 10.1002/gch2.201900024 (PMC6827721; doi:10.1002/gch2.201900024)
Supplement: Supplementary file 1 — Supplementary [file GCH2-3-1900024-s001.pdf]

Copyright WILEY-VCH Verlag GmbH & Co. KGaA, 69469 Weinheim, Germany, 2019.

Global Challenges
Open Access

Supporting Information

for *Global Challenges,* DOI: 10.1002/gch2.201900024

Palmitic-Acid-Based Hydrophobic Deep Eutectic Solvents for the Extraction of Lower Alcohols from Aqueous Media: Liquid–Liquid Equilibria Measurements, Validation and Process Economics

*Rupesh Verma and Tamal Banerjee**

Supplementary Information

Palmitic Acid based Deep Eutectic Solvent for the Extraction of Lower Alcohols from Aqeous Media : Liquid Liquid Equilibria Measurements, Validation and Process Economics

Rupesh Verma and Tamal Banerjee*

Department of Chemical Engineering, Indian Institute of Technology Guwahati, Guwahati, Assam-781039, India

*Corresponding author:

E-mail address: tamalb@iitg.ac.in (Prof. T. Banerjee)

Tel.: +91-361-2582266; fax: +91-361-2582291

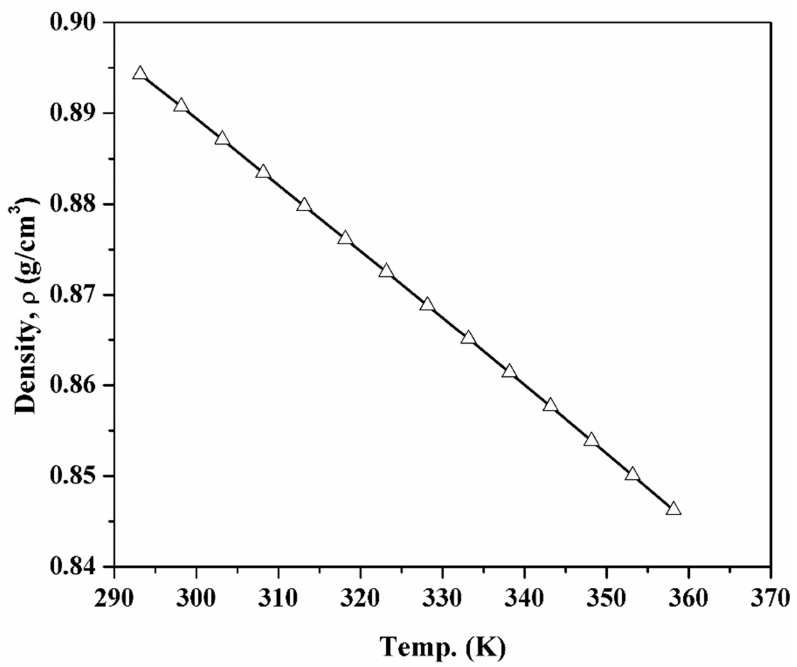

Figure S1. Density of of DL-menthol and Palmitic acid (12:1) based DES at different temperatures

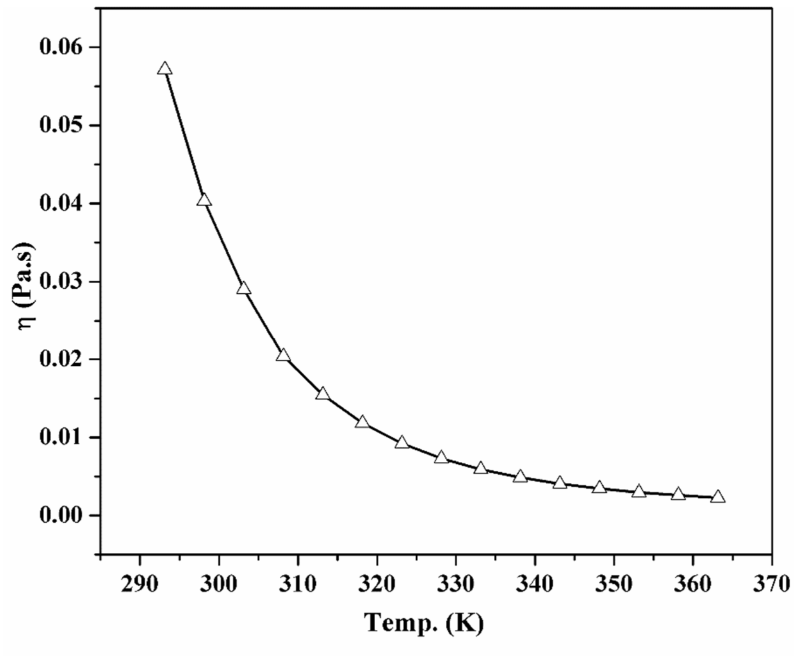

Figure S2. Viscosity of of DL-menthol and Palmitic acid (12:1) based DES at different temperatures

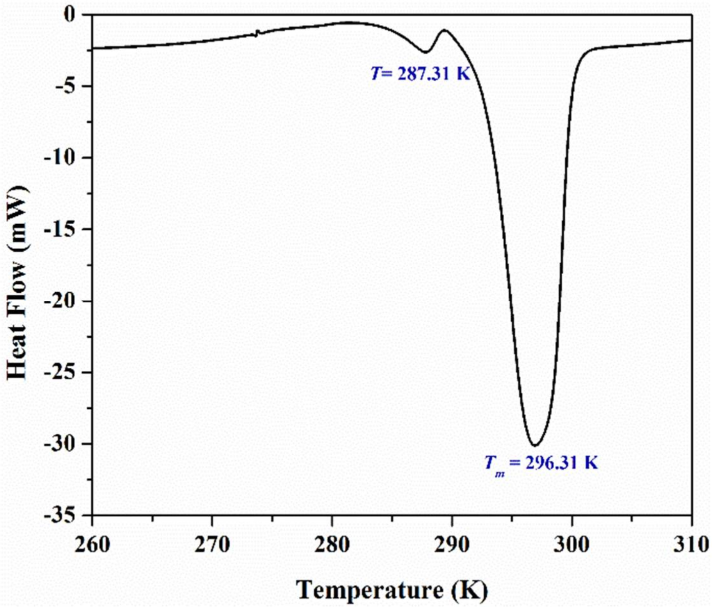

Figure S3. Differential Scanning Calorimetry (DSC) of DL-menthol and Palmitic acid (12:1) based DES

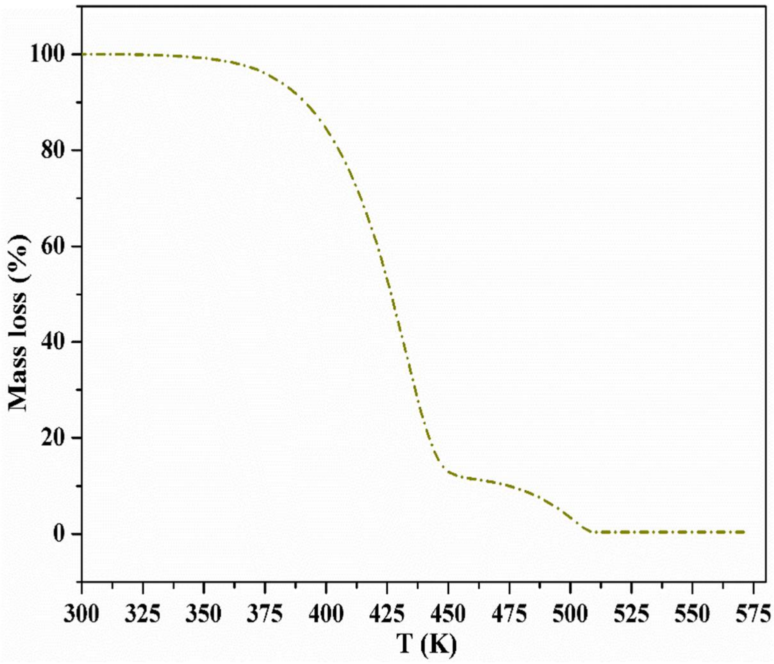

Figure S4. ThermoGravimetric Analysis (TGA) for DL-menthol and Palmitic acid (12:1) based DES

Table S1. Thermal properties of studied eutectic mixtures: Decomposition temperature (T_{dec}) and normal melting temperature (T_m).

Compound Name	T_m (K)	T_{deg} (K)
**Pure compounds**		
DL-menthol	307.15-309.15	309.15
Palmitic acid or Hexadecanoic acid	335.15-339.15	339.15
**Eutectic Mixture**		
DES (DL-menthol:Palmitic acid) 12:1	296.49	541.15

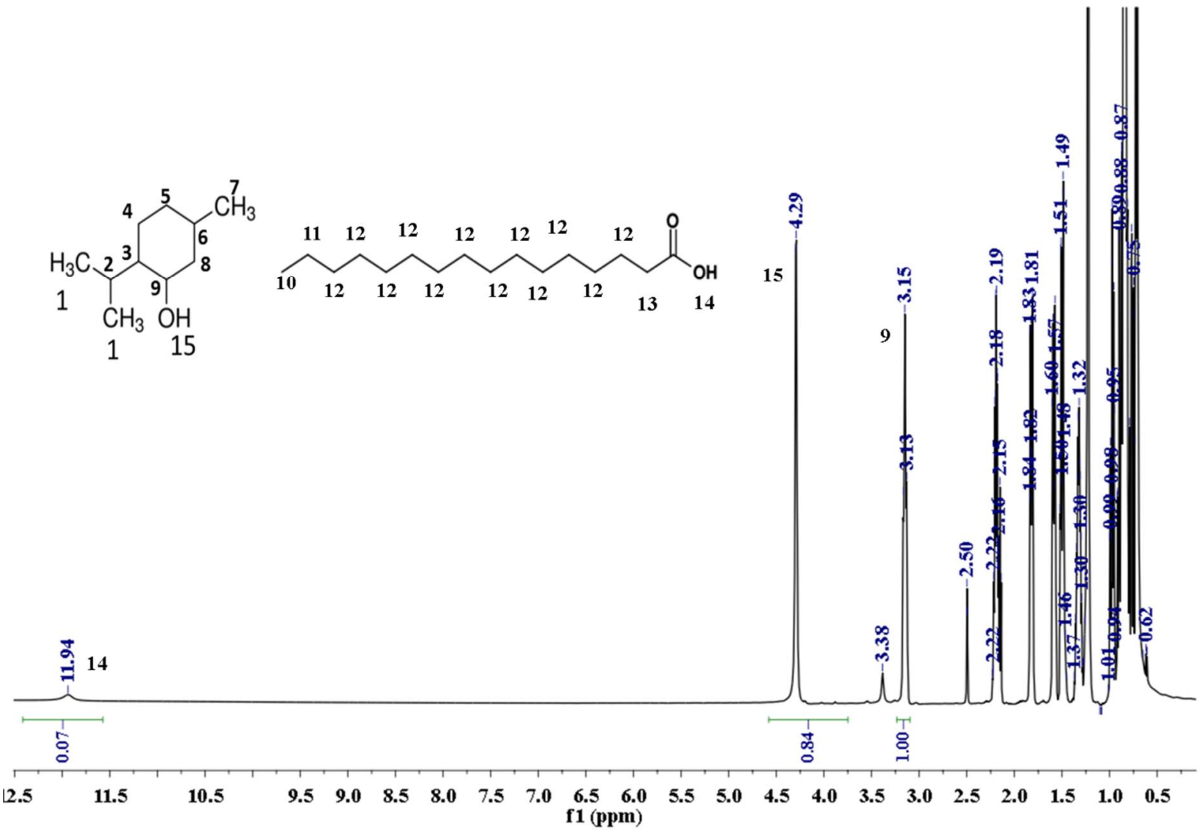

Figure S5. ^{1}H NMR Spectra of Synthesized DL-menthol and palmitic acid based DES

Algebraic Procedure for Computing Mole Fraction in DES-1 from ^{1}H NMR data

**Compound name**	**Corresponding peak**	**Peak Position (PPM)** *	**Corresponding Area**	**Area due to a single atom of H**	**Mole ratio**
DL-menthol	-OH of DL-menthol	4.29	0.84<br><br>(one Hydrogen atoms)	0.84	12
Palmitic acid	-OH of Myristic acid	11.94	0.07<br><br>(one Hydrogen atoms)	0.07	1

*As per Figure S-5

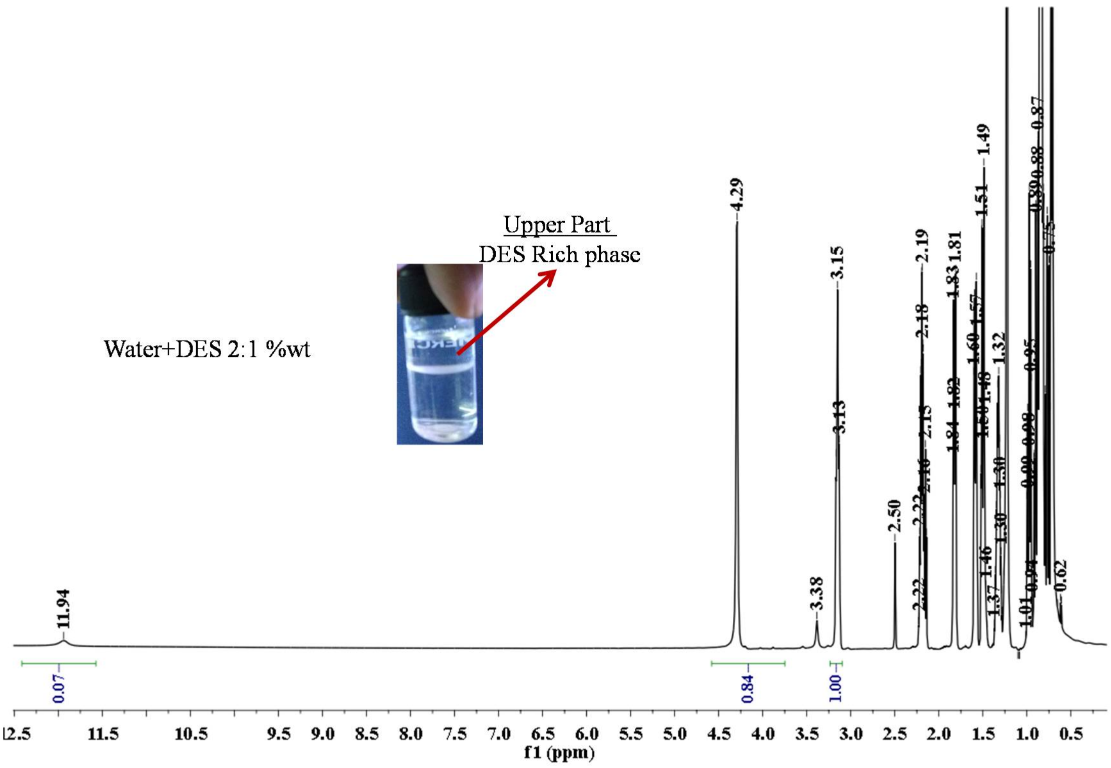

Figure S6. ^{1}H NMR analysis of DES rich phase in water

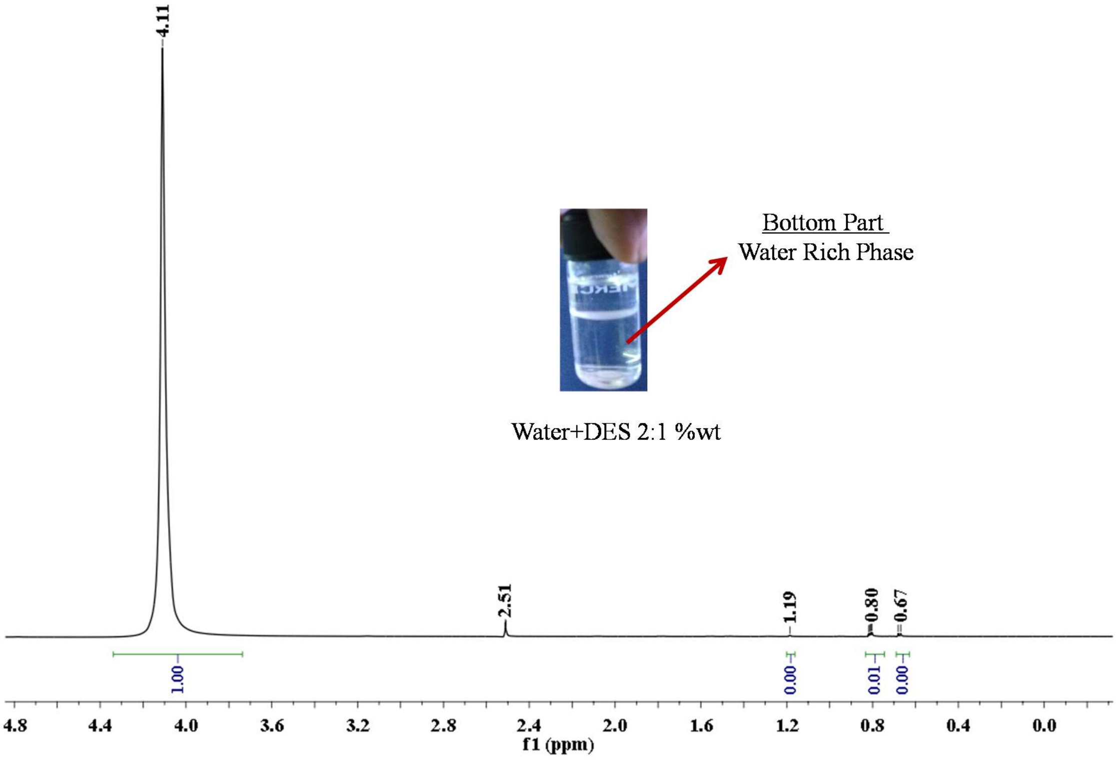

Figure S7. ^{1}H NMR analysis of water rich phase
